# Comparative Study of the Impurity Effect on SnAgCu and SnZn Solder Joints with Electrodeposited Cu

**DOI:** 10.3390/ma17092149

**Published:** 2024-05-04

**Authors:** Yu-Ju Li, Yee-Wen Yen, Chih-Ming Chen

**Affiliations:** 1 Department of Chemical Engineering, National Chung Hsing University, 145 Xingda Rd., South Dist., Taichung 402202, Taiwan; uriath0115@gmail.com; 2 Department of Materials Science and Engineering, National Taiwan University of Science and Technology, 43 Keelung Rd., Sec.4, Da’an Dist., Taipei 106335, Taiwan; ywyen@mail.ntust.edu.tw; 3 Sustainable Electrochemical Energy Development Center (SEED Center), National Taiwan University of Science and Technology, 43 Keelung Rd., Sec.4, Da’an Dist., Taipei 106335, Taiwan

**Keywords:** Kirkendall effect, impurity, electroplated Cu, solders, diffusion

## Abstract

Sn-3Ag-0.5Cu (SAC305)- and Sn-9Zn-based alloys (Sn-Zn-X, X = Al, In) are lead-free solders used in the fabrication of solder joints with Cu metallization. Electroplating is a facile technology used to fabricate Cu metallization. However, the addition of functional additive molecules in the plating solution may result in impurity residues in the Cu electroplated layer, causing damage to the solder joints. This study investigates the impurity effect on solder joints constructed by joining various solder alloys to the Cu electroplated layers. Functional additives are formulated to fabricate high-impurity and low-impurity Cu electroplated samples. The as-joined solder joint samples are thermally aged at 120 °C and 170 °C to explore the interfacial reactions between solder alloys and Cu. The results show that the impurity effect on the interfacial reactions between SAC305 and Cu is significant. Voids are massively formed at the SAC305/Cu interface incorporated with a high impurity content, and the Cu_6_Sn_5_ intermetallic compound (IMC) grows at a faster rate. In contrast, the growth of the Cu_5_Zn_8_ IMC formed in the SnZn-based solder joints is not significantly influenced by the impurity content in the Cu electroplated layers. Voids are not observed in the SnZn-based solder joints regardless of the impurity content, indicative of an insignificant impurity effect. The discrepancy of the impurity effect is rationalized by the differences in the IMC formation and associated atomic interdiffusion in the SAC305- and SnZn-based solder joints.

## 1. Introduction

With the increasing demands for emerging technologies such as electric vehicles, high-frequency communications, and artificial intelligence, the role of the semiconductor industry has become more and more important as these technologies require ultrahigh-performance and ultrafast-speed integrated-circuit (IC) chips. To efficiently integrate multi-functional IC chips within a reduced-volume packaging structure, three-dimensional (3D) IC packaging technology is a promising method to achieve this goal. Copper (Cu)-filled through-silicon-vias (TSVs), filled by electrodeposition, are crucial to constructing a high-efficiency 3D IC packaging structure [1,2,3]. Cu electroplating is a well-established technique and is widely adopted to build interconnections in packaging structures due to many advantages, such as its low cost, low temperature, and relatively simple operation [4]. Organic additives are usually added to the electrolytic solutions to regulate the atomic deposition rates and thereby achieve void-free Cu filling in the TSVs. Traditional additives are inhibitors and accelerators [5,6], and both have a strong adsorption capacity on Cu surfaces, so the co-deposition of additive species with reduced Cu atoms cannot be avoided [6,7].

Soldering is a technique commonly used to construct the interconnects in microelectronic products, using a Sn-based alloy with a low melting point to join two metallization layers on different circuit systems. Due to the low melting point of solder alloys, soldering can be done at relatively low operation temperatures, minimizing thermal damage. In the soldering process, molten solder reacts with the metallization, resulting in the formation of intermetallic compounds (IMCs) at the joint interfaces [8,9,10]. IMC formation is an indicator of successful joints, like the Cu_6_Sn_5_ and Cu_3_Sn phase in Sn-based solder/Cu joints [11,12]. However, the faster growth of IMCs accompanying the formation of Kirkendall voids gives rise to critical reliability issues [13]. The void formation caused by the Kirkendall effect is a well-known phenomenon in Sn-based solder/Cu joints [14,15,16]. Due to the large difference of atomic diffusivities, the Cu atoms diffuse rapidly in the Cu_3_Sn phase rather than the reverse diffusion of Sn into Cu_3_Sn [13]. The out-diffusion of Cu atoms leaves vacancies on the departure sites, while the refilling of vacancies fails due to the slower diffusion rate of Sn. Once the vacancies accumulate to oversaturation, voids are formed at the joint interfaces, mostly at the Cu_3_Sn/Cu interface.

Much literature has reported that the use of organic additives in plating solutions results in the co-deposition of organic species, leaving impurity residues in the Cu electroplated substrate [17,18,19,20,21,22]. When the Cu electroplated substrate contained a high level of impurity residues, serious structural destruction occurred in the Sn-based solder/Cu joints [17,18,19,20,21,22]. The presence of impurities might eliminate vacancy sinks such as grain boundaries and dislocations, accelerating the accumulation and supersaturation of vacancies, and therefore increase the propagation rate of voids in the solder joints [22]. In short, the Kirkendall effect initiates the formation of vacancies, while the impurity effect catalyzes the nucleation and growth of voids by piling up the vacancies. The presence of discontinuous voids provides many fast diffusion paths on the void peripheries, accelerating the atomic interdiffusion as well as the growth of IMCs.

Due to environmental and health concerns, lead-free solders have been extensively used as joint materials in the electronic packaging industry [23,24]. Tin-silver-copper (SnAgCu)-based solders, like Sn-3Ag-0.5Cu (SAC305, in wt.%), are currently representative of the lead-free solders commonly used in consumer electronics, with a moderate melting point (~217 °C) [25]. Nonetheless, this melting point is still 30 °C Celsius higher than that of traditional eutectic SnPb solder, which might cause thermal reliability problems. Alternatively, eutectic Sn-9Zn solder (in wt.%) is a better choice to avoid possible thermal damage problems. Sn-9Zn solder has a lower melting point of 198 °C, closer to that of eutectic SnPb solder (183 °C), which can be better accommodated by existing equipment and materials used for soldering. Material characterizations of Sn-9Zn solder and its interactions with Cu metallization have been performed to offer reliability assessment [26,27]. However, investigations of the impurity effect on the interactions between Sn-9Zn solder and electroplated Cu are rare. It was found that the replacement of SAC305 by Sn-9Zn effectively weakened the impurity effect of electroplated Cu and therefore suppressed the void formation at solder joints subjected to solid-state aging [28]. The void suppression was attributed to the change of dominant IMCs (Cu_5_Zn_8_ instead of Cu_6_Sn_5_ and Cu_3_Sn) and effective substitution of Cu vacancies by Zn [28].

In this study, Sn-9Zn solder and its derivatives (Sn-Zn-X, X = Al, In) were prepared and reacted with Cu electroplated substrates to investigate the impurity effects on the microstructural evolution and void formation at the solder/Cu interface under thermal aging at 120 °C and 170 °C. The organic additives were precisely formulated to adjust the impurity concentrations in the Cu electroplated substrates, thereby controlling the impurity effect. As a comparison, SAC305/electroplated Cu solder joints were constructed and thermally aged to demonstrate the notable impurity effects on the void formation. The results showed that a large number of voids were formed and propagated at the SAC305/Cu interface incorporated with a high impurity content, echoing the findings previously reported [22,29,30]. Accelerated growth of the Cu_6_Sn_5_ phase at the interface incorporated with more impurities was also observed. Reduction of the impurity content to an extremely low level by the precise formulation of plating solutions was helpful to suppress the void formation and IMC growth at the joint interface [22,31]. The impurity effect was insignificant not only in the Sn-9Zn solder joint, as previously reported [28], but also in its derivative systems (Sn-Zn-X, X = Al, In), where no interfacial voids were observed in the aged joints, no matter whether the impurity concentration was high or low. In addition, the IMC growth was also not influenced by the impurities. The absence of interfacial voids was attributed to the deactivation of the Kirkendall effect in the SnZn/Cu system, impeding the formation of vacancies.

## 2. Materials and Methods

The Cu electrodeposition was performed in a Haring cell filling with Cu-containing electrolytes and organic additives. High-purity CuSO_4_·5H_2_O and H_2_SO_4_ were used as the electrolytes to offer the Cu source and to increase the conductivity of the solution, respectively. The additives were formulated by a suppressor, an accelerator, and a promoter, and the formulas are listed in Table 1. Polyethylene glycol (PEG, 50 ppm, 8000 g/mole) was used as the suppressor, bis-(3-sulfopropyl) disulfide (SPS, 2 ppm) was the accelerator, and Cl^−^ (NaCl, 60 ppm) was the promoter. A Cu foil (99.99 wt.%, 160 μm thick, UMAT Co., Hsinchu, Taiwan) with a square area of 1 cm × 1 cm was used as the cathode for the electrodeposition of Cu, and a Cu plate with 0.04 wt.% phosphorus (UMAT Co., Hsinchu, Taiwan) was used as the anode. During the electrodeposition, the current density was set at 3.5 ASD (A/dm^2^) and the solution temperature was controlled at 28 °C. After electroplating for 2 h, the Cu foil at the cathode was removed and the Cu electrodeposited layer was measured to be approximately 50 μm. Two types of Cu electrodeposited layers were fabricated using the additive formulas (Table 1) and the electroplating parameters mentioned above. When the additive formula was PEG and Cl^−^, the Cu electrodeposited layer contained a high concentration of impurity residues (named high-impurity Cu hereafter). Addition of SPS together with PEG and Cl^-^ in the formula produced a Cu electrodeposited layer with a low or even negligible impurity content (named low-impurity Cu hereafter).

Four types of solder alloys were used; one was commercially available SAC305 (Senju Metal Industry Co., Ltd., Tokyo, Japan) and the other three were self-prepared alloys, Sn-9Zn and Sn-Zn-X (where X = 0.45 Al or 5 In, in wt.%). Solder balls weighting 22 mg were prepared and placed on the Cu electroplated layers. Before placing the solder balls, a heat-resistant adhesive tape with an aperture of a diameter of 1.8 mm was applied to the surface of the Cu electroplated layer to control the reaction area between the solder and Cu. The as-prepared solder balls were immersed into a mild etching solution (dilute sulfuric acid) for few seconds to remove surface oxides, followed by rinsing with deionized water. The three types of solder balls were placed on the exposed Cu surfaces, and a tiny drop of flux was applied to the bare Cu surface to prevent oxidation issues. SAC305/Cu, Sn-9Zn/Cu, and Sn-Zn-X/Cu samples were then placed on a hot plate set at 260 °C for 30 s and 230 °C for 180 s, respectively, to perform the liquid solder/solid Cu reactions. After the soldering reactions, the solder/Cu joint samples were placed in an oven set at 120 °C and 170 °C for 0 to 240 h and 0 to 168 h, respectively.

Microstructural examinations of Cu electrodeposited layers and aged solder/Cu joint samples were performed using metallographic methods. For the Cu electrodeposited samples, the surface morphology was observed using a field-emission scanning electron microscope (FE-SEM, UltraPlus, Zeiss, Oberkochen, Germany) and a 2D-probe surface profilometer (DektakXT, Bruker, Billerica, MA, USA). Electron backscatter diffraction (EBSD, Oxford, UK) was used to analyze the grain microstructures, wherein the sample surfaces were treated by electrolytic polishing to smoothen the observed regions. Time-of-flight secondary ion mass spectrometry (TOF-SIMS, ION-TOF, Münster, Germany) was used to obtain the correlation between the depth and impurity distribution in the Cu electrodeposited layers. During the electroplating, the potential change was recorded with the injection of various additives to understand the electrochemical characteristics of the additive molecules. After the soldering and thermal annealing reactions, the solder/Cu joint samples were mounted in epoxy resin, then ground and polished using fine SiC papers and Al_2_O_3_ suspensions, respectively, in a direction perpendicular to the solder/Cu interface. Then, the exposed cross sections of the solder/Cu interfaces were observed using an optical microscope (OM, Olympus BX51, Tokyo, Japan). The FE-SEM, equipped with an X-ray energy-dispersive spectrometer (EDS), was used to analyze the elemental compositions of IMCs formed at the joint interfaces. The average thicknesses of the IMCs were determined by dividing the cross-sectional area of the IMCs by the linear length of the solder/Cu interface.

## 3. Results and Discussion

### 3.1. Effects of Additives on Co-Deposition of Impurities in Electroplated Cu

**Figure 1:**
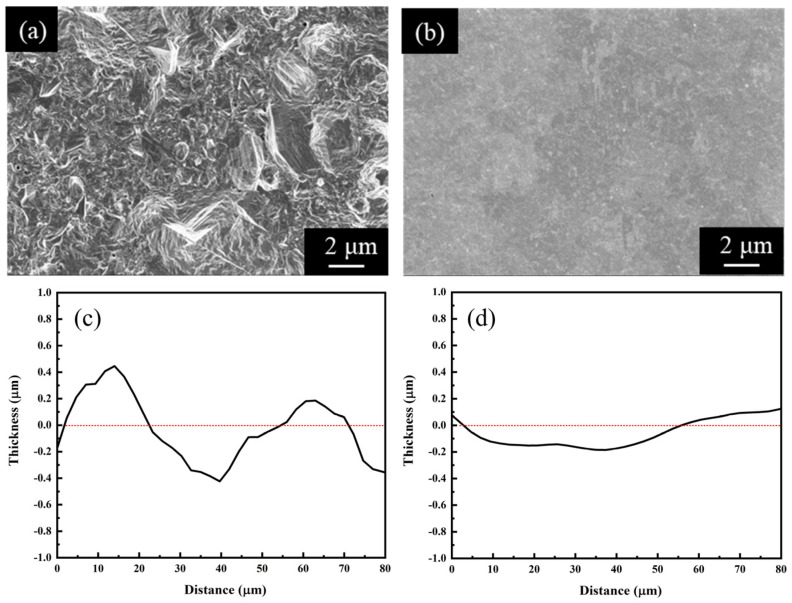
Top-view SEM micrographs and surface profiles of the Cu electroplated layers fabricated using different additive formulas: (**a**,**c**) high-impurity Cu and (**b**,**d**) low-impurity Cu. The thickness at 0 μm in (**c**,**d**) is a baseline, indicating the average thickness of the Cu electroplated layers.

Two different additive formulas were used for the Cu electrodeposition, producing two Cu electroplated layers, high-impurity Cu and low-impurity Cu, as shown in Table 1. Figure 1a,b show top-view SEM micrographs of the two Cu electroplated layers. When the Cu layer (high-impurity Cu) was deposited based on the additive formula of suppressor (PEG) and bridging agent (Cl^−^), its topography was coarse with many bulges, as shown in Figure 1a. When the accelerator (SPS) was added along with PEG and Cl^−^, the resulting Cu layer (low-impurity Cu) exhibited a very smooth surface morphology, as shown in Figure 1b. The surface profiles of the two Cu electroplated layers were also analyzed using a profilometer, as shown in Figure 1c,d, and the results were consistent with the top-view SEM observations. Such a significant morphological change is attributed to the synergistic effect generated by the interactions between the suppressor and accelerator molecules being simultaneously adsorbed on the Cu surface, which regulates the atomic deposition [29,32,33,34].

**Figure 2:**
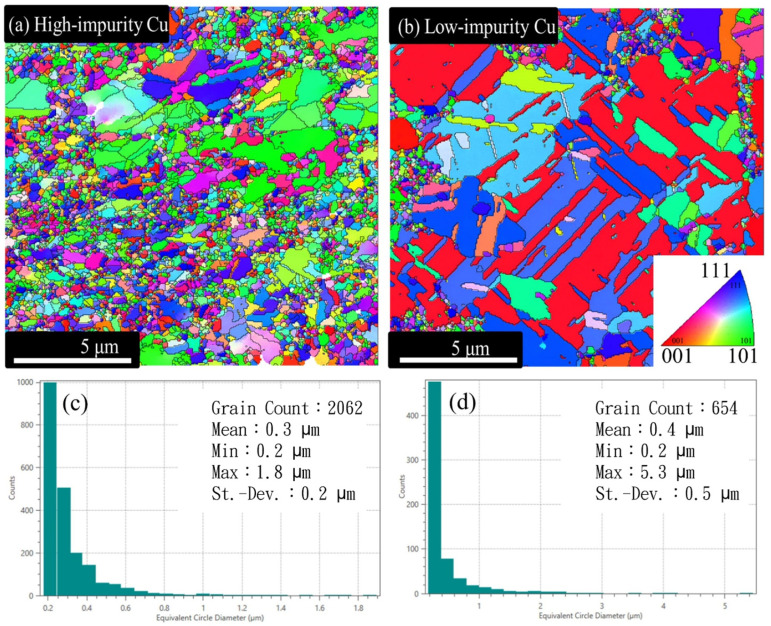
Top-view EBSD orientation maps and grain size distribution histograms of the Cu electroplated layers fabricated using different additive formulas: (**a**,**c**) high-impurity Cu and (**b**,**d**) low-impurity Cu.

Figure 2a,b show top-view EBSD orientation maps of the two Cu electroplated layers. The grain size distribution was not uniform in the two Cu electroplated layers, as shown in Figure 2c,d. The grain size (diameter) of the low-impurity Cu is visually much larger than that of the high-impurity Cu. The grain size in the low-impurity Cu sample can be as large as 5.3 μm, but the maximum grain size in the high-impurity Cu sample is only 1.8 μm. The grain size increment was attributed to the acceleration effect of SPS on the atomic deposition as well as the grain growth. Note that many tiny grains were embedded in the vicinities of giant grains in the low-impurity Cu sample, and therefore the average grain size of the low-impurity Cu sample was slightly larger than that of the high-impurity Cu sample.

**Figure 3:**
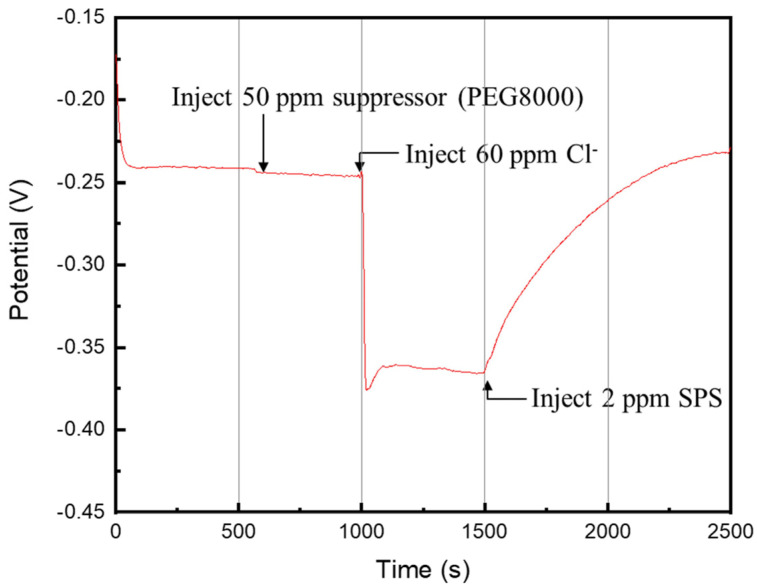
Galvanostatic measurements of the additive injection with PEG, Cl^−^, and SPS, where the molecule weight of PEG is 8000 g/mol.

Figure 3 shows the results of galvanostatic measurements at a constant current, which can be used to understand the efficacy of different additive molecules on Cu deposition. When only a suppressor (PEG) was injected into the plating solution in the early stage (around 600 s), the potential for the Cu electrodeposition remained unchanged. However, an abrupt drop of the potential was observed after an injection of Cl^−^ in the middle stage (1000 s), indicating that the PEG molecules must co-work with the Cl^−^ ions to elaborate the inhibitory effects [35,36]. When SPS was subsequently injected into the plating solution in the later stage (1500 s), the potential gradually increased, showing that the SPS molecules were adsorbed onto the Cu surface, replacing a certain proportion of PEG and accelerating the atomic deposition [22].

**Figure 4:**
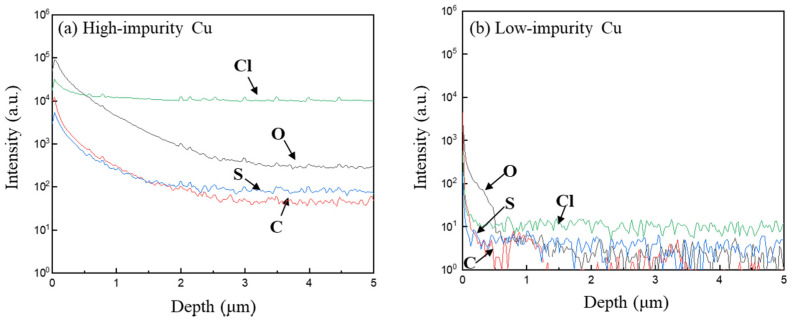
Intensity-depth profiles of the impurity species incorporated in the two Cu electroplated layers: (**a**) high-impurity Cu, (**b**) low-impurity Cu.

Figure 4 shows the SIMS results of intensity-depth profiles of possible impurities incorporated in the two Cu electroplated layers. Four impure substances, carbon (C), sulfur (S), oxygen (O), and chloride (Cl^−^), were detected, as they are the main components of the additives (PEG, SPS, Cl^−^) and acid electrolytes (H_2_SO_4_ and CuSO_4_). As shown in Figure 4, the impurity intensities in the high-impurity Cu sample were at least two to four orders of magnitude higher than those in the low-impurity Cu sample. In addition, the distribution of impurities exhibited an increasing profile for carbon, sulfur, and oxygen from the interior of the Cu layer to the vicinity of the Cu surface. The increasing profiles indicate that the three impure species might segregate to the Cu surface during the grain growth induced by self-annealing at room temperature. Nevertheless, chloride-related species were almost immobile in the Cu electrodeposited layer, which might be attributed to strong interaction between chloride and Cu atoms. The impurity intensities in the low-impurity Cu sample are nearly negligible as they approach the lowest detection limits of SIMS. In other words, the additive formula combining PEG, Cl^−^, and SPS at specific concentrations can effectively suppress the inclusion of impurities in the Cu electroplated layer.

### 3.2. Molecular Adsorption Mechanisms of Additive Molecules

Two types of additive formulas were used to manufacture the Cu electroplated layers, one (PEG + Cl^−^) produced the high-impurity Cu and the other (PEG + Cl^−^ + SPS) produced the low-impurity Cu (with a negligible content of impurity residues). During the Cu electrodeposition process, PEG molecules can interact with Cu+ and Cl^−^ to form a composite layer that adsorbs on the cathode surface, serving as a barrier layer to inhibit the diffusion and reduction of Cu ions [37]. The action of Cl^−^ ions is like a bridging agent, which can help the composite layer adsorb on the cathode surface. Once the composite layer cannot desorb immediately, co-deposition of additive molecules or their fragments occurs, resulting in a large amount of impurities remaining in the Cu electroplated layer [21]. The addition of an effective accelerator, like SPS, even at a concentration much lower than that of PEG, produces a competitive adsorption effect [38,39,40]. The disulfide or mercaptan part of SPS has a high affinity to the cathode surface, so the SPS molecules can adsorb easily onto the cathode, competing with PEG and weakening the adsorption strength of the PEG [39]. The adsorption and activation of SPS was concentration dependent and the activated SPS showed a strong inhibitory effect on the adsorption of PEG [41]. The synergistic effect of competitive adsorption between the suppressor (PEG) and accelerator (SPS) suppresses the co-deposition of impurities, producing a Cu electrodeposited layer with negligible impurity residues (low-impurity Cu).

### 3.3. Impurity Effect on the Microstructural Evolution of the SAC305/Cu Solder Joint

**Figure 5:**
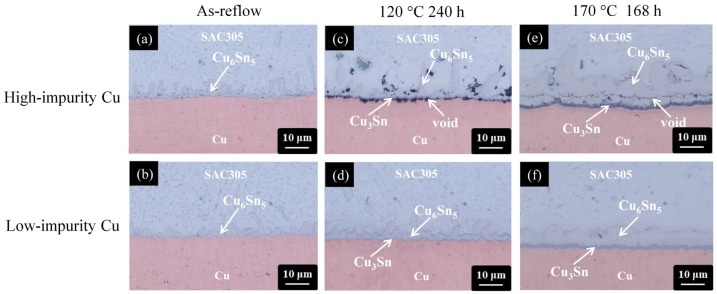
Cross-sectional OM micrographs of the SAC305/Cu interfaces subjected to various treatments: (**a**,**b**) as-reflow, (**c**,**d**) thermal aging at 120 °C for 240 h, and (**e**,**f**) thermal aging at 170 °C for 168 h. (The Cu electroplated layers in the first row and second row are high-impurity Cu and low-impurity Cu, respectively).

To investigate the impurity effect on the solder joints, the two Cu electroplated layers (high-impurity Cu and low-impurity Cu) were joined with different solder alloys, SAC305, Sn-9Zn, and Sn-Zn-X, and then were thermally aged. Figure 5 shows the cross-sectional OM micrographs of the SAC305/Cu interface after thermal aging at 120 °C and 170 °C for 240 h and 168 h, respectively.

**Figure 6:**
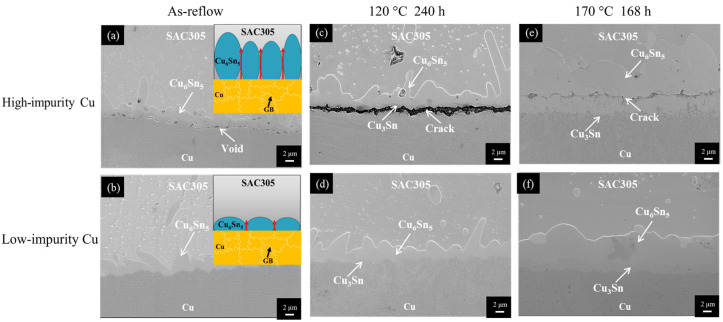
Cross-sectional SEM micrographs of the SAC305/Cu interfaces subjected to various treatments: (**a**,**b**) as-reflow, (**c**,**d**) thermal aging at 120 °C for 240 h, and (**e**,**f**) thermal aging at 170 °C for 168 h. (The Cu electroplated layers in the first row and second row are high-impurity Cu and low-impurity Cu, respectively).

Regardless of the Cu electroplated samples, a thin IMC layer was formed at the SAC305/Cu interface after reflow reaction (as-reflowed), as shown in Figure 5a,b. Based on the compositional analysis of EDX (Cu of 56.1 at.% and Sn of 43.9 at.%), the IMC layer was identified as the Cu_6_Sn_5_ phase [42]. After thermal aging at 120 °C and 170 °C, a new IMC was formed at the Cu_6_Sn_5_/Cu interface and it was identified as the Cu_3_Sn phase based on the EDX results (73.4 at.% Cu and 26.6 at.% Sn). However, the microstructural evolution of the SAC305/Cu interface was different for the two Cu electroplated samples. For the high-impurity Cu sample, voids were formed and propagated along the interfaces, forming a continuous crack, as shown in Figure 5c,e. To clearly observe the void/crack formation, the joint samples in Figure 5c,e were further examined using SEM. From the color contrast shown in Figure 6, the formation of the void/crack at the interface was confirmed. For the low-impurity Cu sample, no voids or cracks were observed at the SAC305/Cu interface subjected to thermal aging at 120 °C and 170 °C, as shown in Figure 5d,f.

The notable distinction shown in Figure 5 indicates a significant impurity effect in the SAC305/Cu system. Voids were formed massively and propagated to form a crack at the joint interface when the Cu substrate contained a high level of impurity residues (high-impurity Cu). As shown in Figure 5c,e, the location of the voids/crack was at the IMC/Cu interface when the aging temperature was 120 °C, but moved upward to the interior of the IMC layer when aging at 170 °C. The formation of the voids/crack and their movement are highly associated with the change of atomic diffusion behavior between Sn and Cu caused by the change of aging temperature, which has been explained previously [21,43]. When the aging temperature is 120 °C, Cu is the dominant diffusion species, governing the growth of IMCs at the SAC305/Cu interface. The out-diffusion of Cu leaves vacancies on the departure side. These additional vacancies can be eliminated by migrating to the vacancy sinks (grain boundaries or dislocation) or accumulate to form voids. Once the vacancy sinks are eliminated by the incorporation of impurities, like the case in the high-impurity Cu, the latter will be accelerated and therefore voids are formed at the IMC/Cu interface. When the aging temperature was increased to 170 °C, the voids/crack formed inside the IMC layer, as shown in Figure 5e. The moving direction of the voids/crack from the Cu side to the solder side implies that the diffusion of Sn also contributes to the growth of IMCs. It was speculated that the diffusivity of Sn was increased when increasing the aging temperature, and with the diffusion of Sn entering the Cu side, new IMC was formed underneath the voids and encapsulated these voids in the IMC layer. The IMC structure became loose due to the void formation, providing fast diffusion paths to promote the interdiffusion of atoms, and therefore the Cu_6_Sn_5_ phase grew at an abnormally fast rate. Compared to the low-impurity Cu, the use of high-impurity Cu to construct the solder joint suffers accelerated void formation and faster IMC growth, exhibiting poor microstructural stability.

### 3.4. Impurity Effect on the Microstructural Evolution of the Sn-9Zn/Cu, Sn-8.55Zn-0.45Al/Cu, and Sn-9Zn-5In/Cu Solder Joints

**Figure 7:**
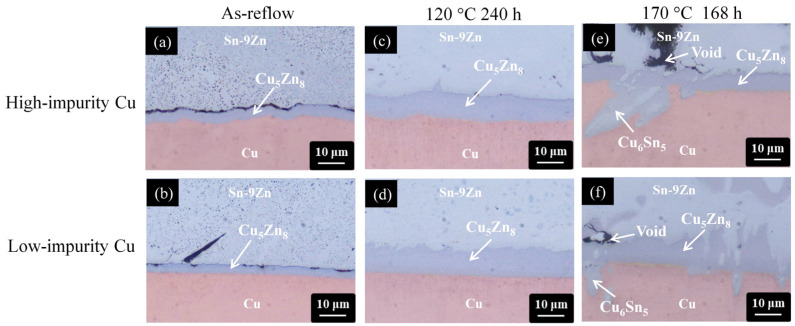
Cross-sectional OM micrographs of the Sn-9Zn/Cu interfaces subjected to various treatments: (**a**,**b**) as-reflow, (**c**,**d**) thermal aging at 120 °C for 240 h, and (**e**,**f**) thermal aging at 170 °C for 168 h. (The Cu electroplated layers in the first row and second row are high-impurity Cu and low-impurity Cu, respectively).

Figure 7 shows a series of cross-sectional OM micrographs of the Sn-9Zn/Cu interface after thermal aging at 120 °C and 170 °C for 240 h and 168 h, respectively. Regardless of the Cu electroplated samples, a thin IMC layer was formed at the Sn-9Zn/Cu interface before thermal aging (as-reflowed), as shown in Figure 7a,b. Based on the compositional analysis of EDX (Cu of 38.7 at.%, Zn of 59.7 at.%, Sn of 1.6 at.%), the IMC layer was identified as the Cu_5_Zn_8_ phase [44]. After isothermal aging at 120 °C for 240 h, the Cu_5_Zn_8_ phase thickened but no voids were observed at the interfaces on the two Cu electroplated samples, as shown in Figure 7c,d. When the thermal aging was performed at 170 °C for 168 h, the Cu_5_Zn_8_ phase started to fracture partially, detached from the interface, and migrated toward the solder matrix due to its brittle nature, as shown in Figure 7e,f [44]. The partial fracture of Cu_5_Zn_8_ generated some channels for the downward diffusion of Sn to the Cu side, and therefore a new IMC, identified as Cu_6_Sn_5_, was formed at the Cu_5_Zn_8_/Cu interface [45,46]. The microstructural characterizations of the Sn-9Zn/Cu interface subjected to isothermal aging are analogous for the high-impurity and low-impurity Cu samples, revealing that the impurity effect is insignificant.

Recalling the impurity effect in the SAC305/Cu system mentioned above, the presence of impurity species eliminated the vacancy sinks in the high-impurity Cu sample, accelerating the vacancy oversaturation to form voids (Figure 5). This phenomenon was not observed in the Sn-9Zn/Cu system (Figure 7). Different from the SAC305/Cu system, the primary IMC formed in the Sn-9Zn/Cu system was the Cu_5_Zn_8_ phase rather than the Cu_6_Sn_5_ phase when the solder joints were thermally aged. The formation of Cu_5_Zn_8_ involves the diffusion of Zn from the solder matrix to the joint interface, followed by the interaction between Zn and Cu. Zn is the minor element in the Sn-Zn alloy matrix and precipitates in the form of dispersed needles. Nevertheless, Zn is more active than Sn and governs the growth of Cu_5_Zn_8_ at the interface. In other words, the vacancies were formed at a faster rate inside the solder matrix due to the departure of Zn atoms. It was speculated that the vacancies were efficiently eliminated by a large number of vacancy sinks (grain boundaries and dislocations) within the Sn matrix before they started to accumulate. Although Cu can also diffuse into the Sn matrix interstitially, the vacancies left on the Cu side can be substitutionally filled by the Zn atoms due to their similar atomic sizes [28]. Further, due to the high solubility of Zn in Cu [47], the Zn atoms have a high affinity for occupying the vacancy or interstitial sites of Cu. As a result, the vacancies cannot efficiently accumulate, and the voids are unable to form. This condition hardly takes place in the SAC305/Cu system because Sn has a relatively low solubility in Cu [48].

**Figure 8:**
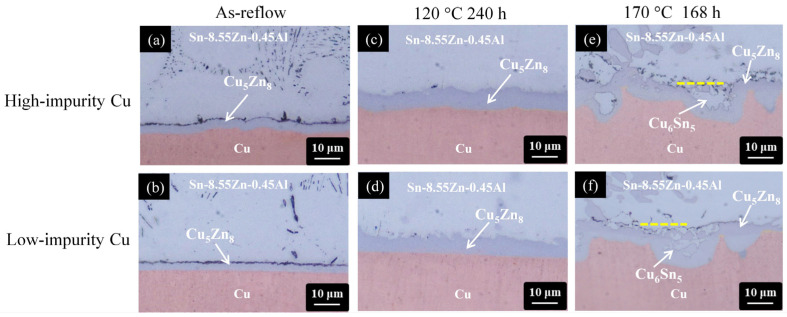
Cross-sectional OM micrographs of the Sn-8.55Zn-0.45Al/Cu interfaces subjected to various treatments: (**a**,**b**) as-reflow, (**c**,**d**) thermal aging at 120 °C for 240 h, and (**e**,**f**) thermal aging at 170 °C for 168 h. (The Cu electroplated layers in the first row and second row are high-impurity Cu and low-impurity Cu, respectively).

Due to the improvement in antioxidation ability, the Sn-Zn-Al alloy system with the addition of Al has been investigated [49,50]. Figure 8 shows a series of cross-sectional OM micrographs of the Sn-8.55Zn-0.45Al/Cu interface after thermal aging at 120 °C and 170 °C for 240 h and 168 h, respectively. Similar to the Sn-9Zn/Cu system, the Cu_5_Zn_8_ phase was formed at the interface after reflow reaction, as shown in Figure 8a,b.

**Figure 9:**
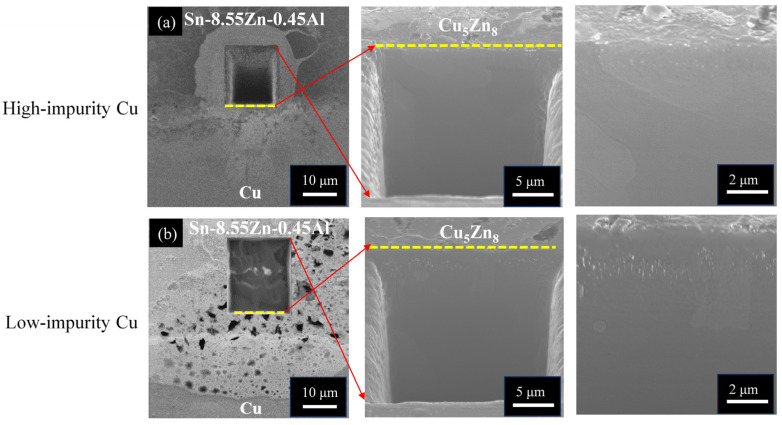
Cross-sectional FIB micrographs of the Sn-8.55Zn-0.45Al/Cu interfaces subjected to thermal aging at 170 °C for 168 h: (**a**) high-impurity Cu and (**b**) low-impurity Cu.

The addition of Al in the solder alloy did not influence the IMC formation due to its very low incorporation (Al of 0.06 at.%, Cu of 37.52 at.%, Zn of 61.88 at.%, Sn of 0.54 at.%). After thermal aging at 120 °C for 240 h, the Cu_5_Zn_8_ phase grew thicker and retained a compact layer structure, as shown in Figure 8c,d. When the aging condition became harsh (170 °C for 168 h), the Cu_5_Zn_8_ phase started to fracture, as shown in Figure 8e,f. The Sn atoms went through fractured paths to reach the Cu side and reacted with Cu to form Cu_6_Sn_5_ underneath the Cu_5_Zn_8_ phase, or embedded inside, like that in the Sn-9Zn/Cu system. Because the microstructural evolution at the Sn-8.55Zn-0.45Al/Cu interface was analogous for the high-impurity and low-impurity Cu samples, the impurity effect is also insignificant, as in the Sn-9Zn/Cu case. Nevertheless, many small voids/cracks were observed at the solder side, near the solder/Cu_5_Zn_8_ interface, as seen in Figure 8e,f. To confirm whether the formation of voids/cracks was caused by mechanical polishing or imbalanced atomic diffusion (Kirkendall effect), the samples in Figure 8e,f were further polished using FIB. Non-destructive ion milling was performed along the yellow dotted line marked in Figure 8e,f to expose the interior microstructure underneath the cross sections. Figure 9a,b shows the resulting FIB micrographs of Figure 8e,f, respectively. The exposed interior exhibited a compact microstructure without the presence of voids and cracks, indicating that the voids/cracks in Figure 8e,f were formed due to mechanical polishing. 

**Figure 10:**
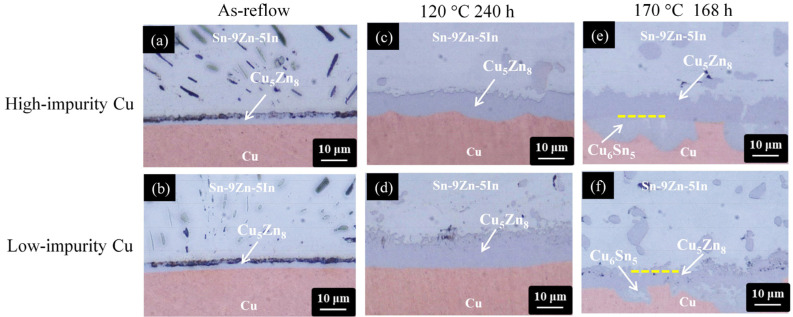
Cross-sectional OM micrographs of the Sn-9Zn-5In/Cu interfaces subjected to various treatments: (**a**,**b**) as-reflow, (**c**,**d**) thermal aging at 120 °C for 240 h, and (**e**,**f**) thermal aging at 170 °C for 168 h. (The Cu electroplated layers in the first row and second row are high-impurity Cu and low-impurity Cu, respectively).

The addition of In (5 wt.%) in the Sn-9Zn alloy can reduce the melting temperature from 198 °C to 188 °C, only 5 °C higher than that of eutectic SnPb solder, so the Sn-9Zn-5In alloy is a potential Pb-free solder [51]. In addition, the addition of In can also improve the wetting and mechanical properties [27]. Figure 10 shows a series of cross-sectional OM micrographs of the Sn-9Zn-5In/Cu interface after thermal aging at 120 °C and 170 °C for 240 h and 168 h, respectively.

**Figure 11:**
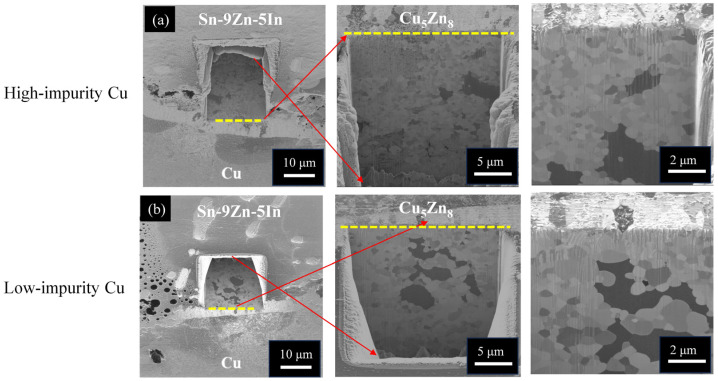
Cross-sectional FIB micrographs of the Sn-9Zn-5In/Cu interfaces subjected to thermal aging at 170 °C for 168 h: (**a**) high-impurity Cu and (**b**) low-impurity Cu.

The microstructural evolution history of the Sn-9Zn-5In/Cu system subjected to thermal aging was analogous to those of the Sn-9Zn/Cu and Sn-8.55Zn-0.45Al systems. Additionally, the interior microstructure of the Cu_5_Zn_8_ phase, shown in Figure 11a,b, exhibited a compact fine-grained structure. More importantly, no voids were observed. Therefore, the impurity effect on the Sn-9Zn-5In/Cu interfacial reactions is insignificant. As previously mentioned in the Sn-9Zn/Cu system, due to the lack of the Kirkendall effect, no additional vacancies were created and therefore no voids could be formed.

### 3.5. IMC Growth in the SAC305 and SnZn-Based Solder Joints

**Figure 12:**
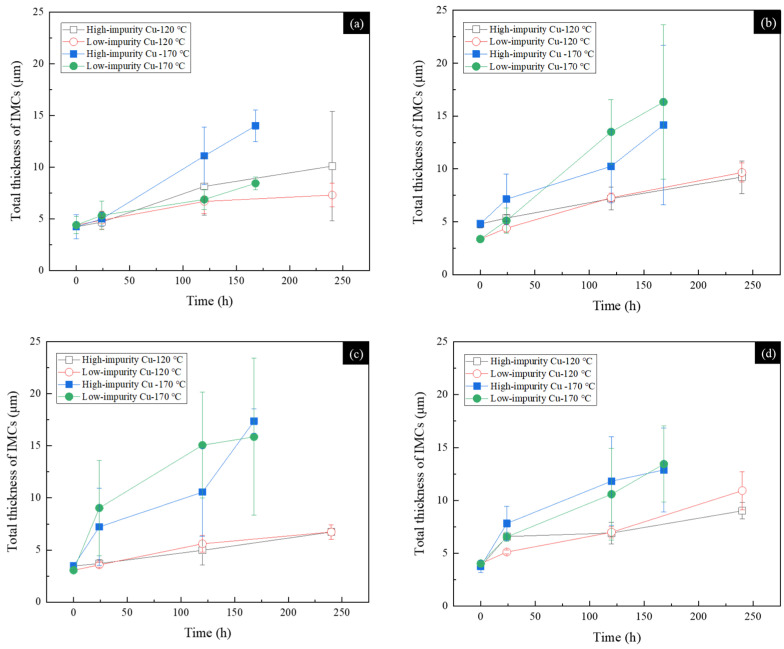
Total thicknesses of IMCs as a function of the thermal aging time for various solders reacted with high-impurity Cu and low-impurity Cu under thermal aging at 120 °C and 170 °C: (**a**) SAC305, (**b**) Sn-9Zn, (**c**) Sn-8.55Zn-0.45Al, and (**d**) Sn-9Zn-5In.

The relationship between the total thicknesses of the IMCs formed in the SAC305 solder joints and the aging time is depicted in Figure 12. In Figure 12a, the Cu_6_Sn_5_ phase grows at a faster rate in the high-impurity Cu solder joints subjected to thermal aging at 120 °C and 170 °C.

Recalling the microstructural observation of SEM in Figure 6, voids were formed massively at the SAC305/Cu interface incorporated with a high impurity content. Intuitionally, the atomic interdiffusion should be impeded by the presence of voids at the SAC305/Cu interface, resulting in the sluggish growth of IMCs. The growth behavior of IMCs depicted in Figure 12a, however, exhibited a trend contradictory to the intuitive inference. It is plausible that the grain morphology of Cu_6_Sn_5_ plays an important role in the accelerated growth behavior. As seen in Figure 6a, the Cu_6_Sn_5_ grains formed in the high-impurity Cu sample after the reflow reaction exhibited a columnar structure. In contrast, the Cu_6_Sn_5_ grains formed in the low-impurity Cu sample exhibited a normal scallop-like structure, as shown in Figure 6b. The difference of grain morphology is more obvious in the low-magnification OM micrographs (Figure 5). As shown in the insets in Figure 6, there are plenty of channels between the columnar Cu_6_Sn_5_ grains in comparison with those between the scallop-like Cu_6_Sn_5_ grains. These channels, as marked by red arrows, served as fast diffusion paths which could accelerate the atomic interdiffusion, and therefore the growth of IMCs was enhanced in the high-impurity Cu samples. The formation of columnar Cu_6_Sn_5_ grains in the high-impurity Cu sample is an interesting phenomenon, but the mechanism is unclear and needs further investigation.

For the SnZn-based solder joints shown in Figure 12b–d, the growth rate of the Cu_5_Zn_8_ phase was analogous in the high-impurity and low-impurity Cu samples subjected to isothermal aging at 120 °C. When the aging temperature was increased to 170 °C, the growth rate of the Cu_5_Zn_8_ phase was slightly different in the high-impurity and low-impurity Cu samples. In the Sn-9Zn and Sn-8.55Zn-0.45Al solder joints, as shown in Figure 12b,c, the Cu_5_Zn_8_ phase grew at a faster rate in the low-impurity Cu sample than in the high-impurity Cu sample. In the Sn-9Zn-5In solder joint as shown in Figure 12d, the Cu_5_Zn_8_ phase in the high-impurity Cu sample grew at a rate similar to that in the low-impurity Cu sample. Overall, the comparison was not consistent in these three SnZn-based solder joints. Considering the difficulty of the thickness measurement of Cu_5_Zn_8_ caused by the IMC fracture, the thickness difference shown in Figure 12b–d was a result of the uncertainty in the measurement. In addition, the Cu_5_Zn_8_ phase was formed as a layer structure after the reflow reaction in both the high-impurity and low-impurity Cu samples. The grain structure of Cu_5_Zn_8_ was distinct from that of Cu_6_Sn_5_ (column or scallop-like grain structure, shown in Figure 6). Due to the lack of fast diffusion channels, the atomic interdiffusion in the SnZn-based solder joints was not influenced by the grain structure as well as the growth of Cu_5_Zn_8_.

## 4. Conclusions

Four solder alloys, SAC305, Sn-9Zn, Sn-8.55Zn-0.45Al, and Sn-9Zn-5In, were joined to two Cu electroplated layers (high-impurity Cu and low-impurity Cu) to investigate the impurity effect on the IMC formation and microstructural evolution at the solder joints aged at 120 °C and 170 °C. The impurity contents in the Cu electroplated layers were successfully controlled by careful formulation of the additive molecules in the plating solutions. In the SAC305/Cu solder joint system, voids were massively formed at the joint interface with the high-impurity Cu sample and the Cu_6_Sn_5_ IMC grew at a faster rate during the thermal aging. The formation of voids was associated with the impurity incorporation in the Cu electroplated layer, accelerating the accumulation of vacancies induced by the Kirkendall effect. In contrast, no interfacial voids were observed in the SAC305/Cu solder joint with a low-impurity Cu sample, indicating a significant impurity effect. In the three SnZn-based solder joint systems (Sn-9Zn, Sn-8.55Zn-0.45Al, and Sn-9Zn-5In), no voids were found in all solder joints no matter whether the Cu electroplated sample was high-impurity or low-impurity. The growth of the Cu_5_Zn_8_ IMCs formed in the SnZn-based solder joints was also not influenced by the impurity content in the Cu electroplated layers. No significant differences were found in the void formation and IMC growth in the three SnZn-based solder joints fabricated with high-impurity and low-impurity Cu samples, indicative of an insignificant impurity effect.

## Figures and Tables

**Table 1:** Two Cu electroplated layers fabricated using different additive formulas.

Sample	Suppressor (PEG)	Bridging Agent (Cl^−^)	Accelerator (SPS)
High-impurity Cu	50 ppm	60 ppm	--
Low-impurity Cu	50 ppm	60 ppm	2 ppm

## Data Availability

Data are contained within the article.
